# Evaluating the epizootic and zoonotic threat of an H7N9 low-pathogenicity avian influenza virus (LPAIV) variant associated with enhanced pathogenicity in turkeys

**DOI:** 10.1099/jgv.0.002008

**Published:** 2024-07-09

**Authors:** Joe James, Saumya S. Thomas, Amanda H. Seekings, Sahar Mahmood, Michael Kelly, Ashley C. Banyard, Alejandro Núñez, Sharon M. Brookes, Marek J. Slomka

**Affiliations:** 1 Department of Virology, Animal and Plant Health Agency (APHA-Weybridge), Woodham Lane, Addlestone, Surrey KT15 3NB, UK; 2 WOAH/FAO International Reference Laboratory for Avian Influenza, Animal and Plant Health Agency (APHA-Weybridge), Woodham Lane, Addlestone, Surrey KT15 3NB, UK; 3 Pathology and Animal Sciences Department, Animal and Plant Health Agency (APHA-Weybridge), Woodham Lane, Addlestone, Surrey KT15 3NB, UK

**Keywords:** avian, avian influenza virus, H7N9, host adaptation, L226Q, pathogenicity, transmission, turkey, receptor, sialic acid

## Abstract

Between 2013 and 2017, the A/Anhui/1/13-lineage (H7N9) low-pathogenicity avian influenza virus (LPAIV) was epizootic in chickens in China, causing mild disease, with 616 fatal human cases. Despite poultry vaccination, H7N9 has not been eradicated. Previously, we demonstrated increased pathogenesis in turkeys infected with H7N9, correlating with the emergence of the L217Q (L226Q H3 numbering) polymorphism in the haemagglutinin (HA) protein. A Q217-containing virus also arose and is now dominant in China following vaccination. We compared infection and transmission of this Q217-containing ‘turkey-adapted’ (ty-ad) isolate alongside the H7N9 (L217) *wild-type* (*wt*) virus in different poultry species and investigated the zoonotic potential in the ferret model. Both *wt* and ty-ad viruses demonstrated similar shedding and transmission in turkeys and chickens. However, the ty-ad virus was significantly more pathogenic than the *wt* virus in turkeys but not in chickens, causing 100 and 33% mortality in turkeys respectively. Expanded tissue tropism was seen for the ty-ad virus in turkeys but not in chickens, yet the viral cell receptor distribution was broadly similar in the visceral organs of both species. The ty-ad virus required exogenous trypsin for *in vitro* replication yet had increased replication in primary avian cells. Replication was comparable in mammalian cells, and the ty-ad virus replicated successfully in ferrets. The L217Q polymorphism also affected antigenicity. Therefore, H7N9 infection in turkeys can generate novel variants with increased risk through altered pathogenicity and potential HA antigenic escape. These findings emphasize the requirement for enhanced surveillance and understanding of A/Anhui/1/13-lineage viruses and their risk to different species.

## Introduction

In 2013, the first human infection with a novel reassortant H7N9 avian-origin influenza A virus (IAV) was reported in China [1]. To date, there have been 1568 laboratory-confirmed human cases of H7N9, which include 616 fatal cases, giving a case fatality rate of 39% [2,3], many of which have been associated with human exposure to infected poultry, predominantly at live bird markets [4]. While chickens, pigeons and ducks have all been reported as positive for H7N9, chickens are considered the maintenance host [5,6]. H7N9 achieved enzootic levels in chickens across large parts of China, circulating in the absence of clinical disease as a low-pathogenicity avian influenza virus (LPAIV) [5,6]. After almost 4 years of extensive circulation in China, a high-pathogenicity AIV (HPAIV) H7N9 variant was detected among one of the two major LPAIV H7N9 clades that had evolved during this period [7,8]. Vaccination of poultry against H7N9 commenced in China in 2017 during the fifth wave and achieved apparent success in reducing detections of human infections [9]. However, despite the reduction of zoonotic H7N9 cases [9], this virus has not been eradicated from poultry in China, and sporadic detection in poultry and the environment of farms and live bird markets have continued across different provinces of China (Fujian, Guangdong and Henan) [3], suggesting continued circulation in Chinese poultry. Genetic analysis of H7N9 viruses that continue to circulate following the adoption of vaccination has consistently identified stable haemagglutinin (HA) gene polymorphisms, namely, the change from a leucine (L) to glutamine (Q) amino acid residue at position 217 in the H7 mature peptide (L217Q: L226Q H3 numbering; L235Q: complete protein numbering) [10,11].

Amino acid position 217 is located in the 220-loop of the HA protein-forming part of the IAV receptor binding site. Possession of an L at position 217 has been previously shown to increase the affinity of HA binding to ‘human-like’, α2-6-linked sialic acid (2-6Sia), while a Q at position 217 increases the affinity of binding to ‘avian-like’, α2-3-linked sialic acid (2-3Sia) [12,14]. Thus, the Q217L polymorphism present in wave 1–5 (2013–2017) H7N9 viruses, along with other molecular motifs such as E627K in the basic polymerase 2 (PB2) protein, have been attributed to the zoonotic potential of these viruses [15,16]. These human H7N9 cases have followed a seasonal pattern with five successive winter waves [17]. The fifth wave of H7N9 (2016–2017) attained its widest geographical distribution in China [17], reinforcing earlier concerns about the spread of H7N9 to neighbouring countries [18]. Travel-related human cases have resulted in the detection of H7N9 in Canada and Malaysia [19,20], while confiscated poultry meat smuggled from China to Japan was found to contain H7N9 LPAIV and HPAIV [21]. In addition, H7N9 has been demonstrated to be able to reassort with co-circulating H9N2 viruses, producing novel viruses with altered biological properties [22,23]. Such events may increase the risk of the emergence of infection in non-chicken hosts, including other avian species, although the requirement and role of further adaptation to different bird species are unclear. Experimental investigations of H7N9 LPAIV in poultry have focused almost exclusively on chickens [24]. Turkeys represent a major poultry species and an important food source globally [25], although the turkey sector in China is comparatively low compared to the duck and chicken sectors [25]. In addition, turkeys have been reported as being highly susceptible to a range of different AIV strains, including H7 subtypes [26,27], and both wild and domestic turkeys have been highly affected during the current H5N1 clade 2.3.4.4b H5N1 panzootic [28,29]. We previously investigated the effects of H7N9 infection and transmission in turkeys [24] and, using the prototypical *wild-type* (*wt*) H7N9 LPAIV of human origin (A/Anhui/1/2013), we demonstrated that direct infection of turkeys resulted in onward transmission to contact turkeys. Moreover, we observed an unexpectedly dramatic increase in pathogenicity for an LPAIV, resulting in clinical disease and significant turkey mortality [24]. Disease severity was correlated with the rapid and consistent emergence of the L217Q polymorphism in the HA gene, leading to the proposal that this genetic change may be influencing susceptibility and virulence in this species [24].

Our current study describes the separate inoculation of chickens and turkeys, using both *wt* and turkey-adapted (ty-ad) H7N9 LPAIV isolates. The infection outcomes in both poultry hosts considered the different pathogenesis, infectivity and transmissibility outcomes for the ty-ad and the *wt* viruses. We also explore factors that might impact the infection outcome, including tissue tropism, receptor distribution and replication kinetics in different avian and mammalian cell types. The zoonotic and reverse-zoonotic potentials of the ty-ad variant were also assessed.

## Methods

### Viruses and the generation of a ty-ad virus variant

Two main viruses were used in this study: (i) A/Anhui/1/13 (H7N9), termed wt and (ii) a ty-ad variant of A/Anhui/1/13 (H7N9) termed ty-ad H7N9. The H7N9 *wt* (A/Anhui/1/13 [H7N9]) was a kind gift from Professor John McCauley at the Francis Crick Institute, UK, which had undergone three egg passages (EP3) in 9–11-day-old specific pathogen-free (SPF) embryonated fowls’ eggs (EFEs) [30]. Three further passages in EFEs produced the EP6 stock, which served as the inocula in the *in vivo* experiments (H7N9 *wt*). The EP6 H7N9 was sequenced in its entirety and confirmed as being identical to the original GenBank sequence submission (accession numbers: CY187618–CY187625) except for an amino acid change in the HA, namely, N141D (complete gene numbering). This exact virus stock was used previously to infect turkeys, which led to the accumulation of amino acid substitutions [24]. A single amino acid substitution, L217Q (L226Q, H3 numbering), was consistently identified in the consensus sequence from all contact turkeys analysed [24]. To evaluate the effect of these substitutions, we previously isolated and propagated a virus (ty-ad) in 12-day-old embryonated turkeys’ eggs (ETEs) by inoculation into the allantoic cavity [24]. Whole-genome sequencing (WGS) of the ty-ad isolate revealed only two polymorphisms compared to the *wt* H7N9 virus, both of which resulted in amino acid substitutions, including the L217Q substitution in the HA gene and the T10S substitution in the neuraminidase (NA) gene.

Both viruses were titrated in EFEs to determine the 50% egg infectious dose (EID_50_) [31] and then diluted. For replication with or without trypsin, a prototypical H7N7 HPAIV was used (A/chicken/England/11406/2008 [H7N7]), as previously characterized [32]. For all viruses used to assess replication kinetics in cell culture, the virus titre was determined by plaque assay on Madin-Darby canine kidney (MDCK) cells as described previously using a final concentration of 1 µg ml^−1^ of L-1-tosylamido-2-phenylethyl chloromethyl ketone (TPCK) treated trypsin (SIGMA) [22].

### Animals

For *in vivo* infection and transmission experiments, both high-health status White Holland Turkeys (*Meleagris gallopavo*) and SPF White Leghorn chickens (*Gallus gallus*) were used at 3 weeks of age. Birds were housed on solid flooring covered with straw, with food supplied *ad libidum* and drinking water refreshed daily. High-health status ferrets (*Mustela furo*) between 700 and 1000 g (around 3 months of age) were used in zoonotic and reverse zoonotic experiments. All birds and ferrets were confirmed as serologically negative to H7N9 by hemagglutinination inhibition (HAI) assay using homologous antigens in serum collected prior to infection. Oropharyngeal (Op) and cloacal (C) swab (chickens and turkeys) and nasal wash (ferrets) samples collected prior to infection were used to confirm negativity to IAV M-gene RNA by reverse transcription real-time PCR (rRT-PCR) [23,33].

### Turkey-to-turkey infection and transmission experimental design

Two separate groups of six, 3-week-old turkeys were directly infected (‘donor’, D0) via the ocular–nasal route with 100 µl of PBS containing 1×10^6^ EID_50_ of the *wt* or ty-ad H7N9 viruses. At 1-day post-infection (dpi), six naïve (contact) turkeys (‘first recipient’, R1) were introduced into each group of directly infected turkeys. All turkeys were weighed immediately prior to infection and daily post-infection.

### Intravenous turkey experimental design

The intravenous (IV) inoculation experiment was performed according to the World Organisation for Animal Health (WOAH) protocol for IV pathogenicity index (IVPI) determination [30], using 3week-old turkeys instead of chickens. Two separate groups of ten, 3-week-old turkeys were directly infected via the IV route with 100 µl of sterile saline containing 1×10^6^ EID_50_ of the *wt* or ty-ad H7N9 viruses.

### Chicken infection and transmission chains design

Two separate groups of six chickens were infected via the ocular-nasal route with 1×10^6^ EID_50_ of the *wt* or ty-ad viruses (D0), diluted accordingly from the respective viral stocks in sterile PBS, with 100 µl inoculum administered per bird. At 1 dpi, six naive chickens were co-housed with the directly infected chickens (R1). At 4 dpi, the D0 birds were removed, the bedding and drinking water were replaced and six additional naive chickens were placed in contact with the R1 chickens (‘second recipient’, R2) to establish a chain of transmission. Drinking water and feed were, respectively, replaced and replenished daily in both the chicken and turkey transmission experiments.

### Ferret model of zoonosis and reverse zoonosis

Six ferrets were infected via the intranasal route with 0.5 ml (0.25 ml per nare) of PBS containing 1×10^6^ EID_50_ of the ty-ad virus. The experiment was conducted in two identical blocks. In each block, three ferrets were housed in the middle of three conjoined housing units, in a linear arrangement, with the central unit separated from its two adjacent units by means of a perforated perspex barrier. One ferret from each block (total of two ferrets) was culled at 6 dpi for immunohistochemistry (IHC) analysis. For each ferret, the external nasal tissues were washed with 1 ml of PBS on 2, 3, 4, 5, 6, 7 and 8 dpi; 1 ml of PBS was used to wash the internal nasal cavity on days 2, 4, 6 and 8 dpi, as previously described [22,34]. Blood was collected from all remaining ferrets at the end of the experiment at 14 dpi, for serological testing. Six chickens and six turkeys were housed in the two adjacent cages at three and 5 dpi for 6 h, after which the birds were moved to a new room with a separate pen for each species and group. All birds were swabbed daily and monitored for clinical signs for 14 days from the last exposure (dpe). Blood samples were collected from all birds exposed to ferrets at 14 dpe.

### Sample collection and processing during the avian infection/transmission studies

All turkeys and chickens were swabbed once daily from the Op and C cavities, and swabs were processed into 1 ml of brain heart infusion broth (BHIB) as previously described [24]. Tissue samples were collected from two turkeys and two chickens pre-destined (random allocation), from each D0 group, for culling while apparently healthy on 4 and 6 dpi. All tissue samples were taken into 1 ml of BHIB to achieve around 10% (w/v) for each sample. Blood samples were collected via wing bleeds from live birds during the study and via terminal heart bleeds from surviving birds at the study’s end, with bleed times indicated in the results and relevant figures. Samples of drinking water and faeces were collected from the environment of infected or contact birds and processed, as previously described [35].

### RNA extraction and AIV rRT-PCR

RNA was extracted from swabs, tissues and environmental samples as previously described [24], and the extracted RNA was tested by the M-gene rRT-PCR, which featured the primers and probe designed by Nagy, Vostinakova [33], as described previously, to detect AIV viral RNA (vRNA) [24]. Ct values were titrated against a tenfold dilution series of H7N9 *wt* RNA of a known titre and converted to relative equivalent units (r.e.u.) as described previously [23]. Ct values ≥36 were considered negative for IAV.

### Whole genome sequencing

WGS was undertaken on the RNA extracted from inocula and clinical samples as previously described [24]. Briefly, double-stranded complementary DNA (cDNA) was synthesized using the cDNA synthesis system (Roche, UK) following the manufacturer’s protocols. Quantification of the synthesized cDNA was performed utilizing the fluorescent PicoGreen reagent, and 1 ng of the cDNA was employed as a template for the construction of the sequencing library using the Nextera XT kit from Illumina (Cambridge, UK). Subsequently, the sequencing libraries were processed on a MiSeq instrument (Illumina, Cambridge, UK), generating paired-end reads of 2×75 bases. Genetic analysis was performed using MEGA X (MEGA Software) [36].

### Serology

Serological analysis was undertaken as previously described for the sera of turkey [24] and chicken [23]. Ferret sera were treated with four volumes of receptor-destroying enzyme (APHA Scientific, Weybridge, UK) as previously described [22]. Detection of seroconversion to subtype-specific (homologous) HA antigens was conducted using the HAI assay, using four haemagglutination units of the corresponding H7N9 LPAIV as the antigen [30].

### Replication kinetics and plaque assay

All cells were incubated at 37 °C with 5% CO_2_ unless stated. Replication kinetics were assessed in chicken embryo fibroblasts (CEF), turkey embryo fibroblasts (TEF), duck embryo fibroblasts (DEF), MDCK (canine), CACO2 (human) and NPTr (swine) cells. Cells (80–90% confluency) were used in six-well plates; after removal of media and wash steps with PBS, the cells were infected with 400 µl of virus at an MOI of 0.01. Each plate was then placed in an incubator for 1 h, gently rocking the plate every 20 min. The viral inoculum was then removed, and the cells were washed with PBS. Dulbecco’s Modified Eagle Medium (DMEM) containing TPCK trypsin (SIGMA) with a final concentration of 1 µg ml^−1^ was then added to each well. The plates were incubated, and samples were collected at the indicated time points and stored at −80 °C for further use. For plaque assays, 6-well plates of TEFs or MDCKs were inoculated with tenfold serially diluted virus in DMEM and added to the plates as above. Flu overlay (immunodiffusion grade melted agarose, Sigma-Aldrich) containing TPCK trypsin with a final concentration of 1 µg ml^−1^ was added to each well; 2 ml of this mixture was then added to each well. The plates were inverted to prevent condensation build-up in the wells and incubated for a period of 4 days. Plaques were visualized using crystal violet (Sigma), counted, and p.f.u. ml^−1^ were calculated as previously described [22].

### IHC and characterizing 2-3Sia and 2-6Sia receptor distribution in avian tissues

Tissue samples were fixed in 10% (v/v) neutral buffered formalin (VWR, East Grinstead, UK) and processed routinely through graded alcohols and chloroform and embedded in paraffin wax. Four-micrometre-thick sections, cut on a rotary microtome, were used for IHC detection of influenza A nucleoprotein as previously described [24]. For lectin staining of virus receptor distribution in tissues, slides were dewaxed in xylene and passed through graded alcohols to a Tris-buffered saline solution with 0·05% Tween (TBSt) (0·005M Tris, pH 7·6, 0·85% w/v NaCl). Samples were subsequently incubated with biotinylated *Maackia amurensis* lectin II (MALII) (1/100) (Vector Laboratories, Peterborough, UK) for the 2-3Sia, or biotinylated *Sambucus nigra* (SNA) (Elderberry) bark lectin (1/1000) (Vector Laboratories) for the 2-6Sia for 1 h, and VECTASTAIN Elite ABC-peroxidase reagent (Vector Laboratories) for 30 min, at room temperature. Sections were washed three times with TBSt between incubations. The IHC staining was visualized using 3,3 diaminobenzidine (Sigma-Aldrich, Poole, UK), and sections were counterstained in Mayer’s haematoxylin (Surgipath, Peterborough, UK), dehydrated in absolute alcohol, cleared in xylene and mounted using dibutyl phthalate xylene and glass coverslips. Samples were processed and images captured in a double-blinded fashion and scored semi-quantitatively by trained veterinary pathologists based on the intensity and distribution of the staining (–, absent; +/–, minimal; +, mild; ++, moderate).

### Statistical analysis

To assess disparities among various groups, one-way ANOVA, two-way ANOVA or paired students t-tests were carried out (GraphPad Prism v8). A significance threshold of *P* values (<0.05) was set. Area under the curve (AUC) analysis was performed on total shedding as previously described [35] using GraphPad Prism v8.

## Results

### The ty-ad H7N9 virus has increased pathogenicity for turkeys but not chickens

**Fig. 1.**
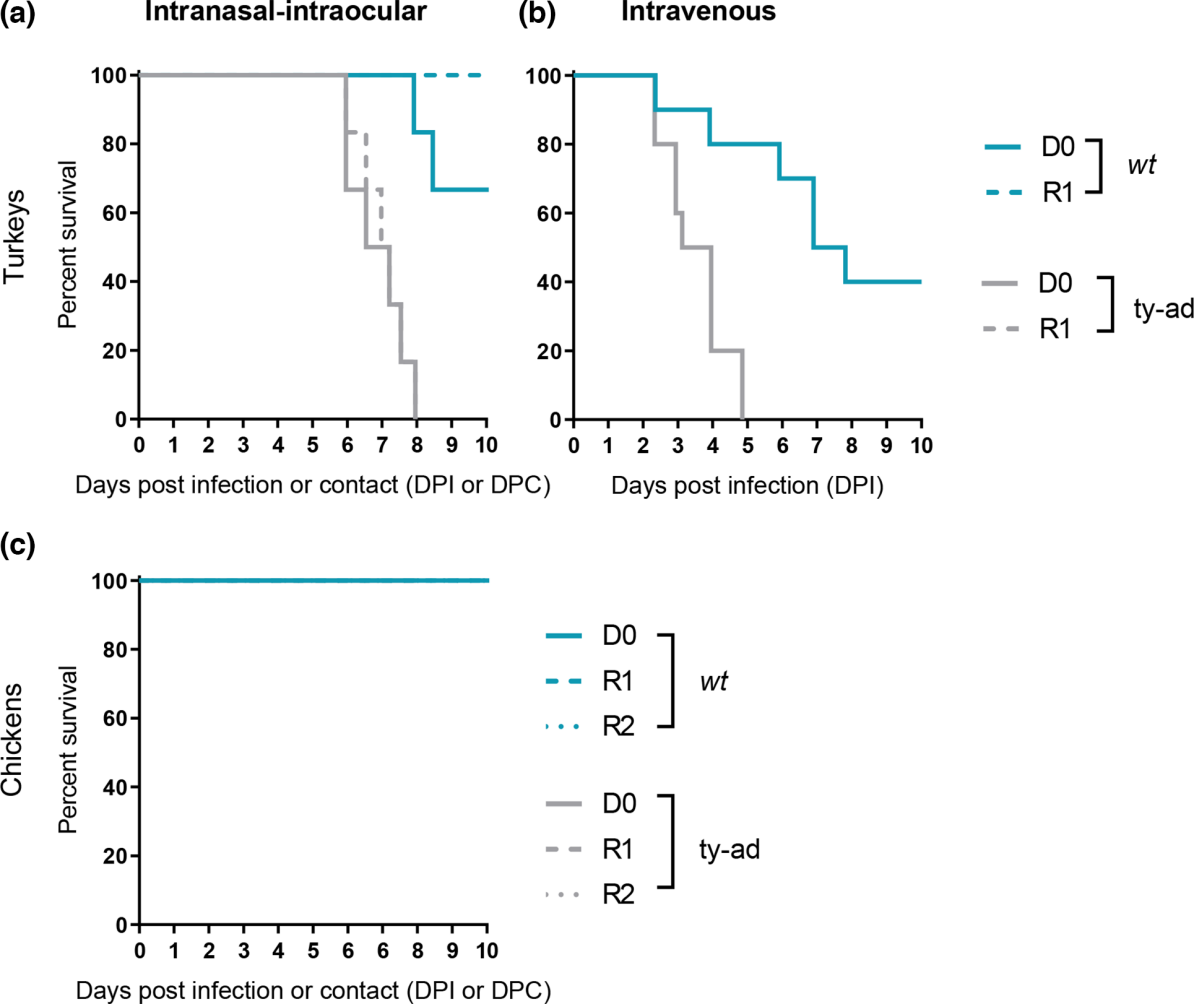
Survival of turkeys and chickens infected with H7N9 *wt* and ty-ad virus variants. (**a) and (c)** The survival rates of six turkeys (**a**) or chickens (**c**) directly inoculated (**D0**) via the intranasal-intraocular route (solid lines) with 1×10^6^ EID_50_ of the *wt* (teal lines) or ty-ad (grey lines) virus variants. The survival of six turkeys (**a**) or chickens (**c**) co-housed with either group at 1 dpi (**R1**) (dashed lines) or six contact chickens added at 4 dpi (dotted lines) is also shown. (**b)** The survival rates of ten turkeys inoculated intravenously with 1×10^6^ EID_50_ of *wt* (teal lines) or ty-ad (grey lines) virus variants.

Both the *wt* and ty-ad H7N9 viruses were previously characterized as LPAIV by standard IVPI testing in chickens [24]. Following direct ocular-nasal infection of turkeys, the *wt* virus caused 33% mortality in the D0 turkeys between 8 and 8.5 dpi (Fig. 1a). Comparatively, the ty-ad virus caused 100% mortality in D0 turkeys between 6 and 8 dpi. Similarly, following transmission to naive contact turkeys, the *wt* virus caused 0% mortality, yet the ty-ad virus caused 100% mortality (Fig. 1a). Where mortality was observed, turkeys typically exhibited mild clinical signs including ruffled feathers and huddling behaviour, during the preceding timepoints. Importantly, all surviving *wt* virus-infected turkeys demonstrated seroconversion to H7N9 when tested by HAI at 14 dpi, which confirmed infection (data not shown). A similar difference in pathogenicity was observed when turkeys were inoculated with either virus intravenously; the *wt* virus caused 60% mortality by 8 dpi, whereas the ty-ad virus caused 100% mortality within 5 dpi (Fig. 1b). The ty-ad virus infected turkeys also exhibited reduced but not statistically significant weight gain compared to the *wt*-infected turkeys, for both D0 and R1 groups (Fig. S1, available in the online version of this article). By contrast, chickens (both D0 and R1 groups) exhibited 0% mortality (Fig. 1c) with either virus. Taken together, these data indicate that the ty-ad virus is more pathogenic than the *wt* virus in turkeys but not in chickens.

### Both the *wt* and ty-ad virus-infected birds shed similar levels of vRNA and transmitted infection equivalently in both turkeys and chickens

**Fig. 2.**
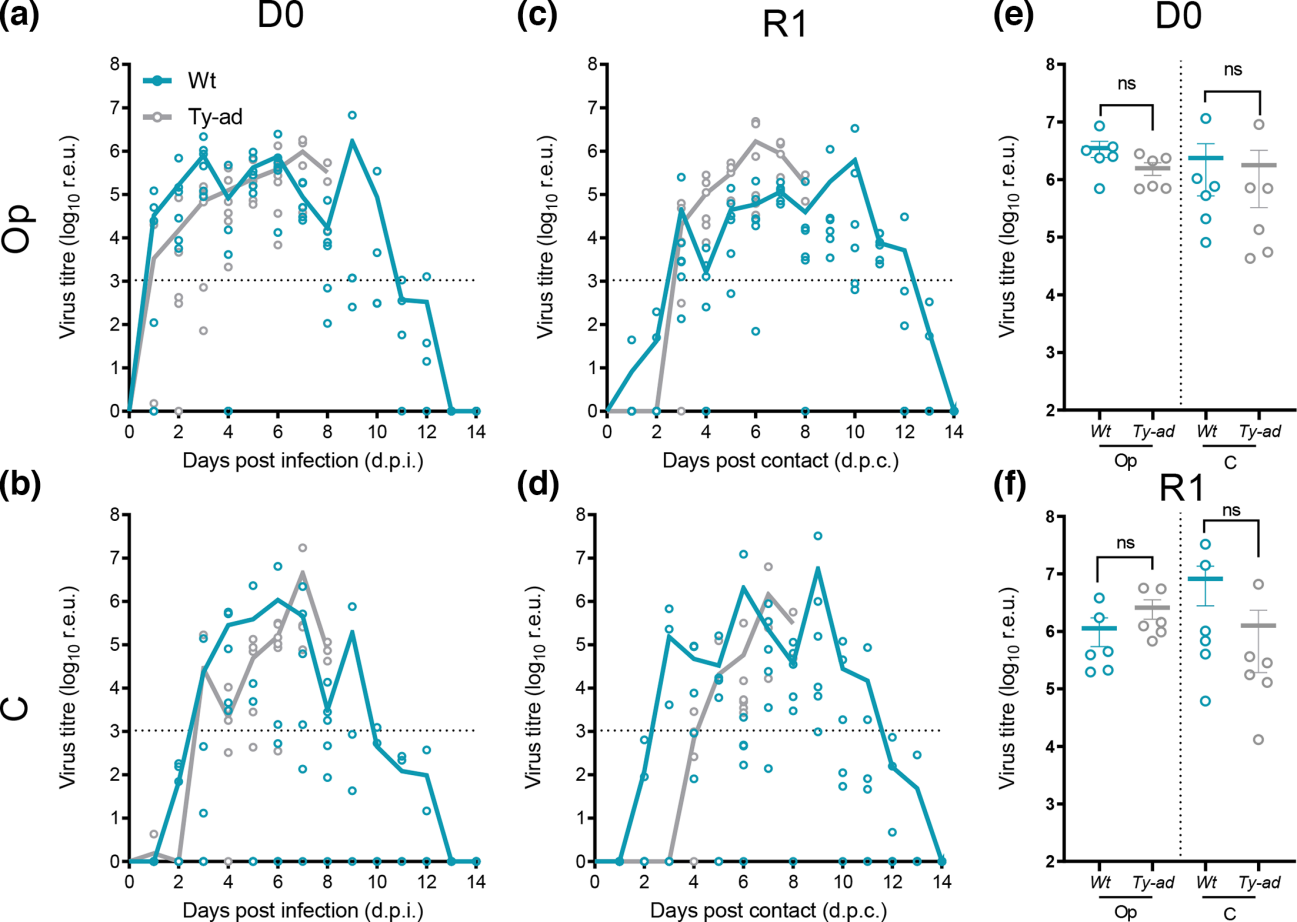
vRNA shedding from *wt* and ty-ad virus-infected or contact turkeys. vRNA shedding profiles from turkeys directly inoculated with 1×10^6^ EID_50_ with *wt* (teal lines) or ty-ad (grey lines) virus variants (**D0**) (a and b) or turkeys co-housed with either group at 1 dpi (**R1**) (c and d). Symbols show individual bird vRNA levels, and lines indicate the mean vRNA levels for all live birds in a given group, quantified as r.e.u. (see Methods for details). vRNA shedding was quantified from swabs collected from the Op (a and c) or C cavities (b and d). Dotted horizontal lines indicate the limit for positivity at 36 ct values (1.05×10^3^ r.e.u). Shedding profiles from individual birds were used to calculate the AUC and graphically displayed for the directly infected (**D0**) (**e**) or contact (**R1**) (**f**) birds. One-way ANOVA with multiple analysis was performed comparing the Op and C AUC shedding for both the D0 and R1 groups, ns (not significant) indicates *P*-value > 0.05.

**Fig. 3.**
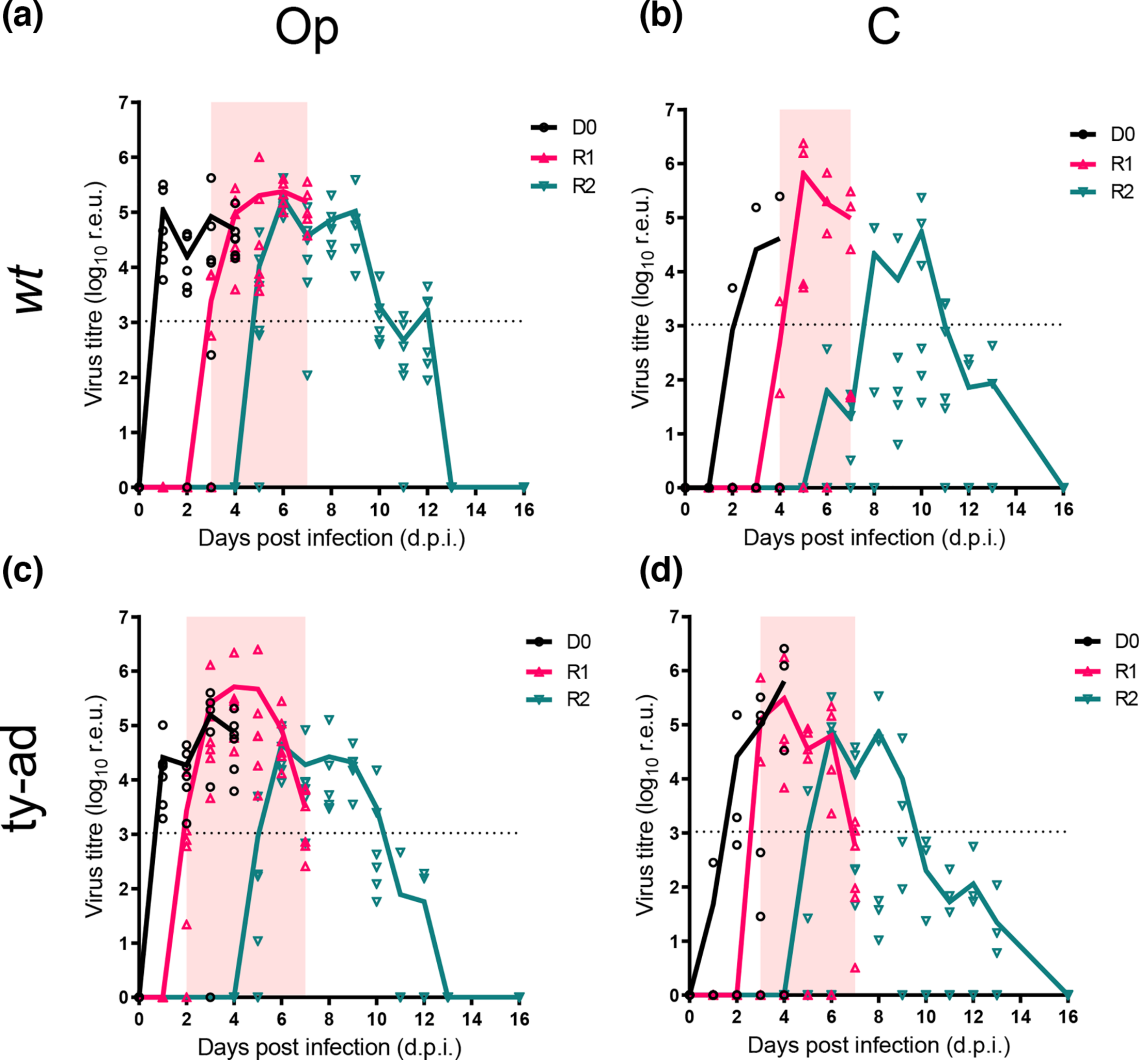
vRNA shedding from *wt* and ty-ad virus-infected or contact chickens. vRNA shedding profiles from chickens directly infected with 1×10^6^ EID_50_ with *wt* (a and b) or ty-ad (c and d) virus variants (**D0**) (black lines) or chickens co-housed with either group at 1 dpi (R1, red lines) or 4 dpi (R2, teal lines). The D0 chickens were removed prior to the addition of second contact (**R2**) chickens at 4 dpi to facilitate an extended transmission chain. Symbols relate to individual birds. and lines indicate the mean virus titre (r.e.u.), the pink shaded areas indicate the window of detectable vRNA being shed from the R1 chickens. Dotted horizontal lines indicate the limit for positivity at 36 ct values (1.05×10^3^ r.e.u.). vRNA shedding was quantified from swabs collected from the Op (a and c) or C cavities (b and **d**).

Both the *wt* and ty-ad viruses successfully infected all six of the respective D0 turkeys or chickens. For both groups, vRNA was detected in both the Op and C swabs taken from turkeys (Fig. 2a and b) and chickens (Fig. 3). Directly infected (D0) turkeys and chickens shed peak levels of vRNA of between 1×10^5^ to 1×10^6^ r.e.u. from the Op cavity following infection with either virus (Figs2a  3a ). Based on AUC analysis, there was no significant difference in the total amount of virus shed from turkeys following infection (Fig. 2e and f). The levels of vRNA detected in the water, and environmental faecal sources, were largely comparable between turkeys and chickens infected with either virus, although greater vRNA was detected in the faeces deposited by the ty-ad group between 4 and 8 dpi (Fig. S2B).

The transmission efficiency was 100% for both viruses in both species; all R1 turkeys and chickens became infected and shed vRNA from the Op and C cavities (Figs 2c & d and 3). For the turkeys, positive vRNA levels were first detected for both *wt* and ty-ad R1 groups at 3 days post-contact, indicating transmission within a similar timeframe. Op and C vRNA shedding profiles for the R1 contact turkeys were similar to those of the directly infected turkeys (Fig. 2a and b compared to C and D). For the chickens, a chain of transmission was established from D0 to R1 and subsequently to R2 chickens, with 100% successful transmission throughout the chain. However, the ty-ad vRNA was detected in swab material from the R1 chickens a day earlier than the *wt* virus (Fig. 3a and c).

All chickens and turkeys that survived until the end of the study (19 dpi) were seropositive by the HAI against the H7N9 antigen (data not shown). Interestingly, using post-infection sera raised against the *wt* or ty-ad viruses, there was a statistically significant drop in reciprocal HAI titres between the wt (L217) and ty-ad (Q217) antigens for heterologous compared to homologous sera, suggesting that aa position 217 may be an antigenically important residue (Fig. S3).

### Differences in systemic H7N9 distribution between turkeys and chickens, with increased tissue dissemination of ty-ad in turkeys compared to *wt* virus

**Fig. 4.**
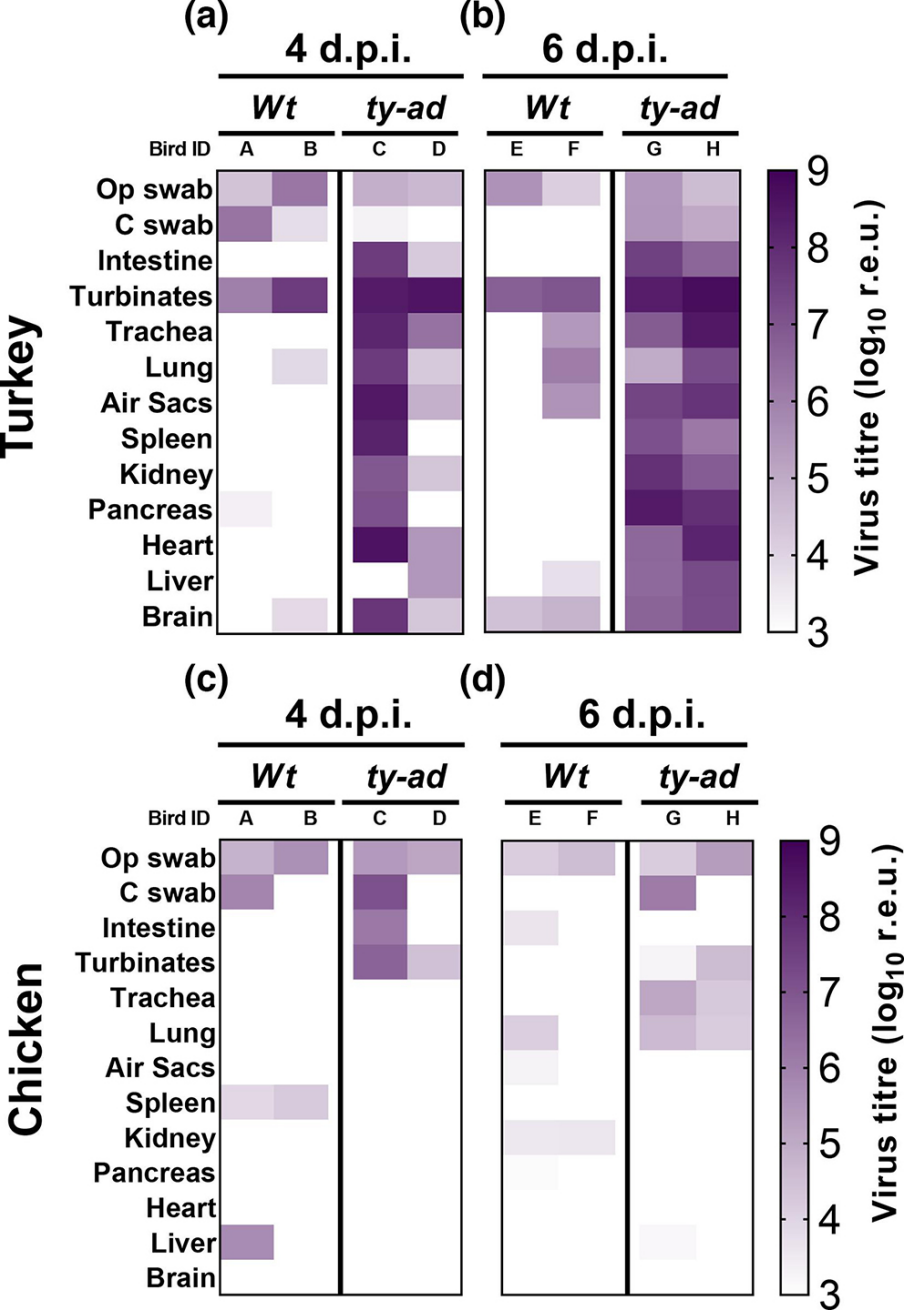
vRNA levels in different organs of directly infected turkeys and chickens. vRNA was quantified in organs collected from turkeys (a and b) or chickens (c and d) directly infected with 1×10^6^ EID_50_ of the *wt* or ty-ad virus variants. Organs were taken from two birds in each group which were culled at 4 dpi (a and c) or 6 dpi (b and d). The detection of vRNA is shown graphically in a heat map ranging from the limit of positive detection at 1.05×10^3^ (pale colour) to 1×10^9^ r.e.u. (dark colour).

The tropism of the *wt* and ty-ad viruses was investigated by vRNA quantification and viral antigen detection by IHC. Tropism was investigated in tissues collected from at least two turkeys and two chickens per group, culled while apparently healthy at 4 and 6 dpi (Fig. 4). In the turkeys, both the *wt* and ty-ad vRNA were detected in a range of organs, including respiratory (nasal turbinates, trachea, lung and air sacs) and other visceral (including the intestine, pancreas, spleen, heart, liver and brain) organs (Figs 4a, b and S4). However, the ty-ad virus was detected more frequently and had higher vRNA and IHC (Table S1) levels than the *wt* virus. By contrast, chickens exhibited considerably lower vRNA levels in all organs, with limited dissemination beyond the respiratory and enteric tracts, at both 4 and 6 dpi (Fig. 4a, b compared to d and e). There were also no major differences in virus tropism between the *wt* and ty-ad viruses in the chickens (Fig. 4c, d, Table S1).

### Turkeys and chickens have slightly different 2-3Sia and 2-6Sia receptor distributions in their respiratory tracts and visceral organs

The L217Q mutation has been previously shown to change virus receptor preference from 2-6Sia to 2-3Sia [12]. The ty-ad virus possesses a Q at amino acid position 217, therefore we hypothesized that differences in 2-3Sia and 2-6Sia might explain the expanded tissue tropism of the ty-ad virus in turkeys. Using lectins that bind to 2-3Sia (MALII) or 2-6Sia (SNA), the host receptor distribution was assessed in turkey and chicken tissues, where the highest levels of vRNA were detected (nasal turbinates, trachea, lung, pancreas and kidney) (Figs 4 and S5, Table 1). Turkey and chicken tissues had similar 2-3Sia and 2-6Sia receptor staining distribution, although some differences were observed. Turkey tissues had more intense MALII (2-3Sia) labelling in the nasal cavity and trachea compared to SNA (2-6Sia) staining (Table 1). Further, in turkeys, staining in the nasal cavity and trachea was more intense for MALII (2-3Sia) compared to that observed in the same tissues from chickens (Table 1). Interestingly, while SNA (2-6Sia) staining in the trachea of turkeys was less pronounced than that seen in chickens, the SNA (2-6Sia) staining in turkey kidneys was more intense than observed in chickens (Table 1).

**Table 1.** 2-3Sia and 2-6Sia distribution in chicken and turkey tissues

Lectin (receptor)	Tissue	Chicken			Turkey			Chicken consensus	Turkey consensus
		A	B	C	A	B	C		
**MAL II(2-3Sia)**	Nasal cavity	+/−	+	+	++	++	++	**+**	**++**
	Trachea	+/−	+	+	++	++	++	**+**	**++**
	Lung	+	+	+	+	+	+	**+**	**+**
	Pancreas	+	+	+	+	+	+	**+**	**+**
	Kidney	++	++	++	++	++	++	**++**	**++**
**SNA (2-6Sia)**	Nasal cavity	+	++	+	+/−	+	+	**+**	**+**
	Trachea	+	++	++	+	++	+	**++**	**+**
	Lung	+	+	+	+	+	+	**+**	**+**
	Pancreas	+/−	+	+	++	+	+	**+**	**+**
	Kidney	+	+	+	++	++	++	**+**	**++**

Semi-quantitative scoring was performed in blinded fashion; −, absent; +/−, minimal; +, mild; ++, moderate.

### Both *wt* and ty-ad viruses require trypsin for efficient replication, but the ty-ad virus replicates to higher titres in avian cells

**Fig. 5.**
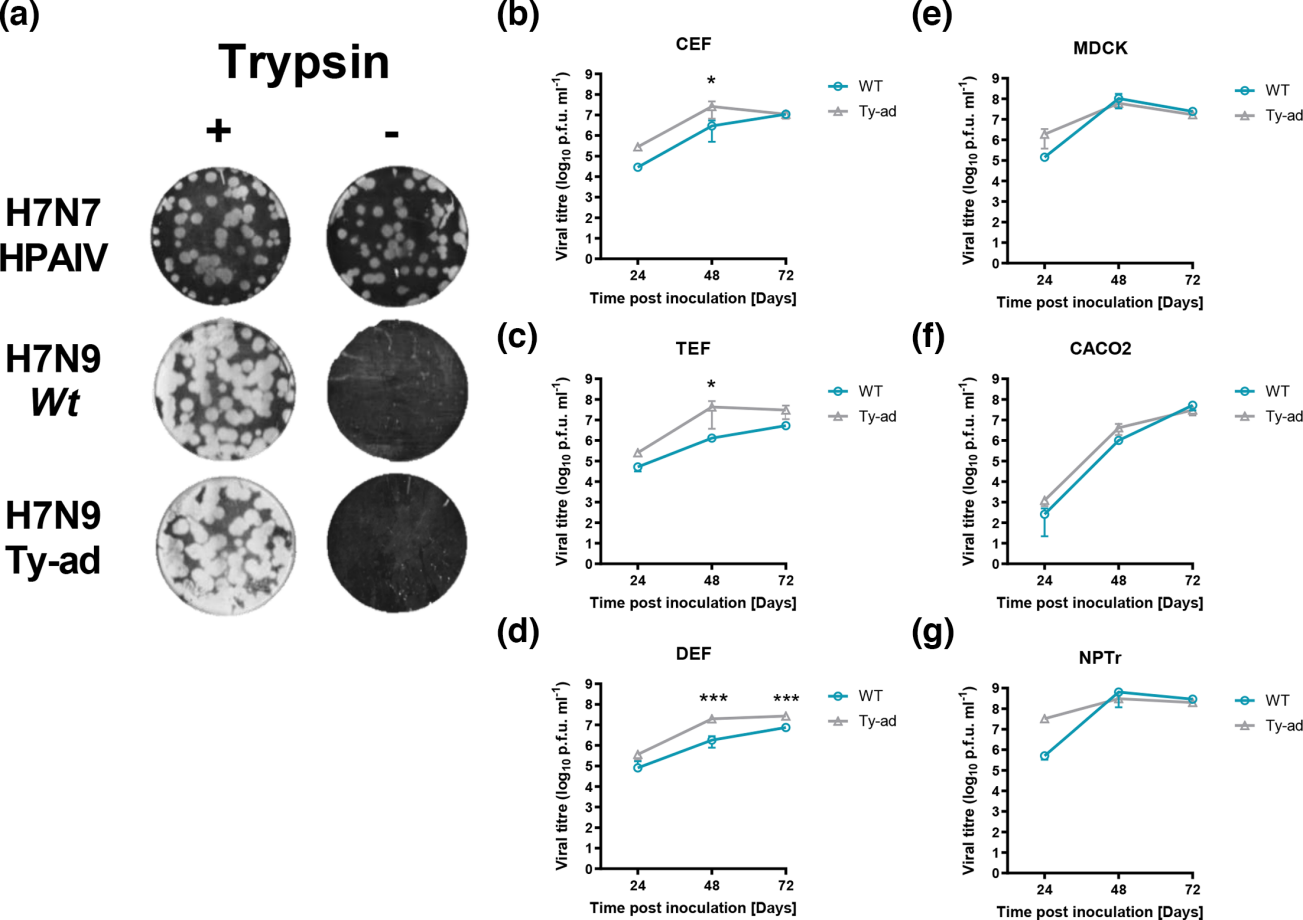
Replication kinetics of *wt* and ty-ad virus variants in cells from different species. (**a)** Representative well-visualizing plaques on TEF cells from three independent replicates with the same dilution of wt, ty-ad or A/chicken/England/11406/2008 (H7N7 HPAIV) viruses in the presence (+) (1 µg ml^−1^) or absence (–) of TPCK trypsin. Plaque assays were stained with crystal violet. (**b–g)** Virus replication of *wt* (teal) and the ty-ad (grey) virus variants in chicken, turkey or duck embryo fibroblasts (CEF, TEF and DEF), MDCK (canine), CACO2 (human) and NPTr (swine) cells. Cells were infected at a MOI of 0.01 with each virus and cell supernatant was harvested at 24, 48 or 72 h post-infection. Viral titres were determined based on plaque-forming units per ml (p.f.u. ml^−1^) using a plaque assay. Each time point corresponds to the mean of four biological replicates with standard deviations indicated. Two-way ANOVA with multiple analysis was performed comparing the two strains, * indicates *P*-value < 0.05, ** indicates *P*-value < 0.005 and *** indicates *P*-value ≤ 0.001.

vRNA from Op swab samples collected at 6 dpi from all six turkeys directly infected with the ty-ad virus was sequenced. Sequence analysis revealed a single basic cleavage site (CS) sequence present in HA (PEIPKGR’GLF) in all sequences, identical to the CS sequence in the wt and ty-ad viruses used for the inocula. In cell culture, both the *wt* and ty-ad H7N9 viruses required the presence of exogenous TPCK trypsin to replicate; both viruses formed plaques when TPCK trypsin was added yet failed to form plaques without TPCK trypsin addition (Fig. 5a). In comparison, H7N7 HPAIV formed plaques even without trypsin (Fig. 5a). The *wt* and ty-ad viruses were also compared for replication in primary duck, chicken and turkey embryonic fibroblast (DEF, CEF and TEF) cells as well as human (CACO-2), swine (NPTr) and canine (MDCK) cells (a cell line highly susceptible and permissive to IAV replication). In all three of the different avian cells, the ty-ad virus replicated to statistically significantly higher titres compared to the *wt* virus (Fig. 5b–d). In the different mammalian cells, however, there was no statistically significant difference in virus replication between the *wt* and ty-ad viruses over the 72 h culture period (Fig. 5e–g).

### The ty-ad virus represents a zoonotic threat but does not exhibit reverse zoonotic transmission under experimental conditions

**Fig. 6.**
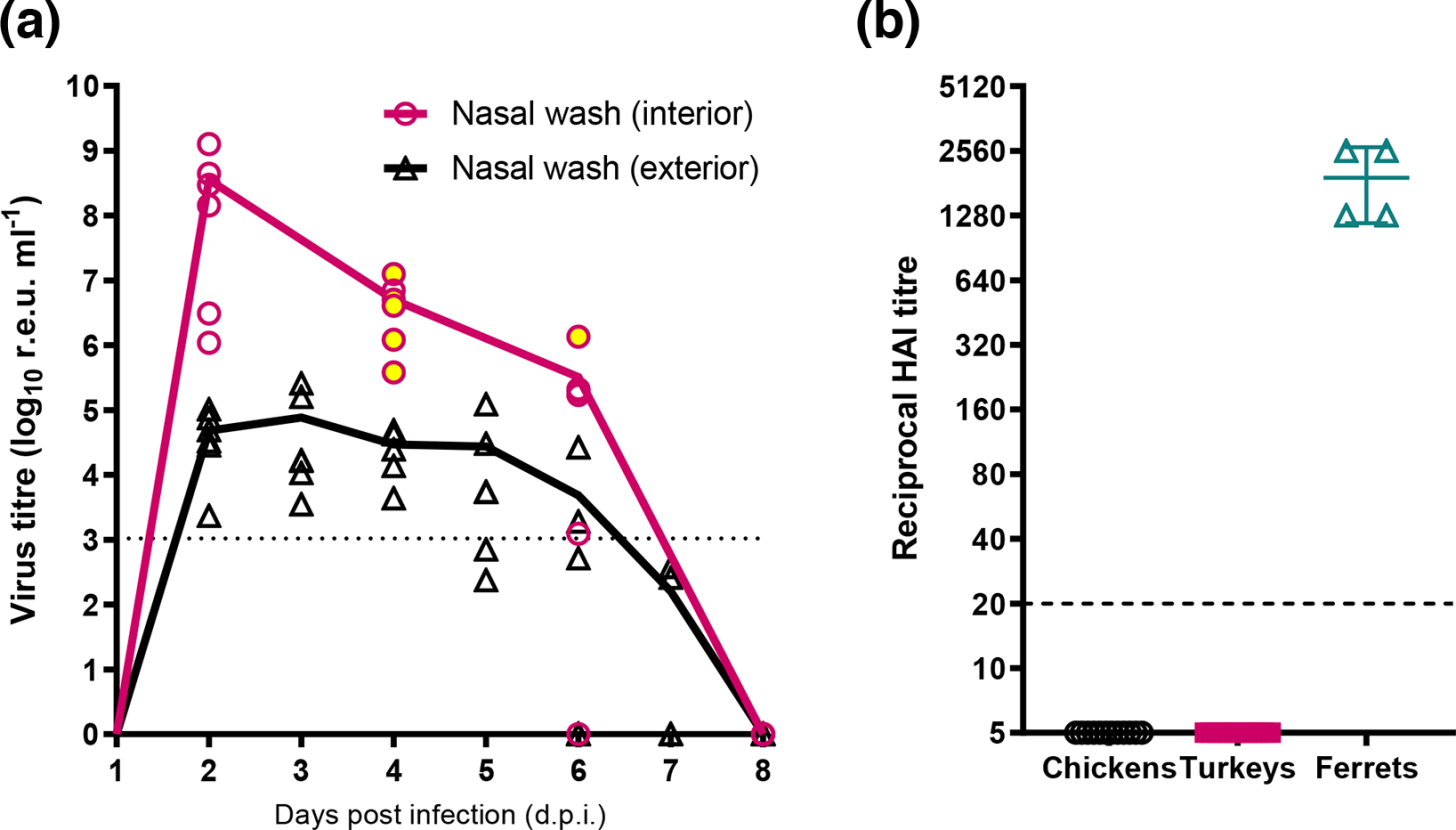
Zoonotic and reverse zoonotic infection dynamics for the ty-ad virus. Infection dynamics of the ty-ad virus in the ferret model and investigation of reserve zoonotic infection from ferrets to chickens and turkeys. (**a)** Virus titre estimates (r.e.u.) derived from interior (pink) or exterior (black) nasal wash samples collected from six ferrets (three per block) directly infected intranasally with 1×10^6^ EID_50_ of the ty-ad virus. One ferret per block (a total of two ferrets) was culled at 6 dpi for IHC analysis. Symbols show an individual ferret’s vRNA levels in the washes, while continuous lines indicate the mean vRNA levels. Yellow-filled circles indicate the interior nasal wash samples for which sequencing of amino acid position 217 in HA was performed and confirmed as Q217 (samples from five ferrets at 4 dpi and one ferret at 6 dpi). The dotted horizontal line indicates the limit for positivity at 36 ct values (1.05×10^3^ r.e.u) (**b)** HAIs were performed on sera taken from four directly inoculated ferrets at the end of the experiment (14 dpi). Chickens (*n* = 12) and turkeys (*n* = 12) were similarly bled at 14 dpi, after having been housed in the same air space, yet physically separated, from the infected ferrets for a 6 h period at 3 dpi. HAI assays were performed using homologous H7N9 antigen. The dashed horizontal line indicates the limit for positivity at a titre of 20 HAI values (1.05×10^3^ r.e.u.).

We next investigated the zoonotic consequences of the ty-ad virus by investigating its ability to infect and replicate in ferrets, a model for human IAV infection. Four ferrets (two per block) were directly inoculated intranasally with 1×10^6^ EID_50_ of the ty-ad virus and successfully became infected, with vRNA being detected in interior nasal wash samples from 2 until 6 dpi (Fig. 6a). vRNA was also detected (above the threshold for assay positivity) from 2 to 6 dpi from exterior nasal wash samples. WGS was performed on the viruses present in the interior nasal wash samples from all ferrets at 4 or 6 dpi (Fig. 6; yellow-filled circles). Sequence analysis revealed that viruses present in all ferrets showed no polymorphisms compared to the input virus at a consensus level (data not shown). Influenza viral NP was also detected in the respiratory turbinates, trachea and lungs of the two ferrets pre-destined for IHC analysis at 6 dpi (Fig. S6 and Table S2). All four of the remaining ferrets that bled at the study’s end (14 dpi) had been seroconverted to homologous H7N9 antigen by HAI (Fig. 6b). To mimic reverse zoonoses, the ability of infected ferrets to transmit viruses to naive turkeys and chickens was investigated. No vRNA was detected in either avian Op or C swabs collected daily for 14 dpe from either block (data not shown). In addition, at 14 dpe, none of the chickens or turkeys had seroconverted to homologous H7N9 antigen by HAI from either block (Fig. 6b).

## Discussion

Since its emergence in 2013 in China, A/Anhui/1/13-lineage H7N9 LPAIV has continued to circulate in poultry, threatening human health. Currently, H7N9 is located exclusively in China [37] where poultry production consists largely of chickens and ducks with almost negligible turkey production [25]. Despite widespread poultry vaccination in China, H7N9 continues to be detected in poultry [3], with an ever-present concern that geographical expansion of H7N9 could increase its zoonotic and epizootic risk through reassortment with indigenous AIVs, or access to novel hosts [22].

Previously, the risk of A/Anhui/1/13-lineage H7N9 infection in turkeys was investigated, modelling a scenario in which the H7N9 virus expands into neighbouring countries with a larger turkey poultry sector [24]. Infection and transmission of H7N9 LPAIV in turkeys were associated with the 217 L217Q (H7 numbering of the mature protein) polymorphism in the HA gene [24]. An H7N9 virus from a turkey with severe clinical disease (termed the ‘ty-ad’ virus) was isolated that contained two amino acid substitutions, one in the HA (L217Q) and one in the NA (T10I) proteins. In this study, we explored the outcome of infection for two gallinaceous poultry species (chicken and turkey) with this ‘ty-ad’ virus variant compared to the *wt* virus. We further investigated potential mechanisms that might underpin the differences in observed infection outcomes and explored the zoonotic and reverse zoonotic risks of these viruses.

*In vivo,* when inoculated intravenously or intranasally, the ty-ad virus caused greater, and more rapid, mortality in turkeys than the *wt* H7N9 virus strain. This disparity in clinical disease between the *wt* and ty-ad viruses was not observed in experimentally infected chickens. Post-mortem investigation of the turkeys in the ‘ty-ad’ virus-infected group revealed a systemic tissue dissemination of vRNA with higher levels of vRNA being detected in a wider range of visceral organs compared to the *wt* virus. By contrast, following chicken infection, this difference in vRNA levels and its systemic distribution was not observed between the two virus variants. However, both viruses displayed comparable shedding profiles from both the Op and C cavities. Different pathogenic outcomes between chickens and turkeys infected with H7 AIVs have been reported previously [38]. However, the genetic determinants for the virulence and pathogenesis of HPAIV in turkeys are largely unknown. For AIVs, tropism is determined by a combination of factors including (i) the distribution of proteases required to cleave HA0 to HA1 and HA2 required for infectivity, (ii) the distribution of the SA receptors to which the viral HA binds and (iii) cells being permissive (allowing viral replication) for viral infection. We therefore explored these factors, which might underpin the observed differences in pathogenicity and tropism between the turkey and chicken hosts following infection with the two H7N9 LPAIV variants.

The major genetic determinant of AIV tropism is the presence of multiple basic amino acids proximal to the CS in the HA protein, termed a multibasic cleavage site (MBCS). This MBCS facilitates the cleavage of HA0 to HA1 and HA2 (required for infectivity) by proteases found ubiquitously in animal tissues [39]. Sequence analysis of the *wt* and ty-ad virus inocula confirmed a single basic CS typical of an LPAIV. Moreover, both viruses required TPCK trypsin for growth in turkey cells (TEFs), affirming an LPAIV phenotype. Therefore, the expanded tropism of the ty-ad virus in turkey tissues may be independent of the restriction imposed by the tissue localisation of proteases, which typically restricts the tropism of most LPAIVs to mainly the respiratory and/or enteric tracts of chickens and turkeys [39]. Proteolytic activation of LPAIV HA occurs extracellularly, driven by airway proteases, the trypsin-like serine proteases human airway trypsin-like protease and transmembrane protease serine 2, although a minority of viruses possess HA CS motifs that are processed by other proteases (reviewed by Kido, Okumura [39]). These ‘atypical’ viruses, including the 1918 IAV [40], can utilize proteases found ubiquitously in tissues of multiple organs for cleavage, and therefore can potentially increase tissue dissemination and pathogenicity. For H9N2 infections, changes in the CS have been shown to alter the clinical outcome in turkeys [41]. The predominant proteases present in other cell types and how they may differ in distribution in turkeys compared to chickens, along with which proteases may differentially cleave the HA of the *wt* and ty-ad viruses, remain to be identified. However, conserved expression and functionality of the furin protease have been observed between chickens and ducks [42]. Therefore, considering that turkeys and chickens are closely related galliformes, with ~90% genome identity [43], it is unlikely that notably separate protease compositions exist between the two species. However, non-basic amino acid variations in H9N2 viruses have been shown to bestow an HPAIV phenotype in turkeys compared to chickens [41]. However, the L217Q substitution, present in the HA of the ty-ad virus, is not located near the HA1/HA2 CS and is therefore unlikely to alter the protease preference.

The L217Q polymorphism has been previously shown for various IAV strains including H7N9, to change the receptor preference by increasing the affinity of HA to 2-Sia [12,14]. The A/Anhui/1/13 isolate being a human-derived virus has a naturally high affinity for 2-6Sia, but is also able to bind 2-3Sia with a relatively high affinity [14,44,46]. Recent receptor preference analyses for H7N9 have demonstrated that Q217 increases affinity to 2-3Sia [14]. Consequently, we investigated whether the expanded tropism could be due to a greater density of 2-3Sia in turkey tissues. In this study, we did not see any difference in pathogenicity between the wt and ty-ad viruses in chickens, we therefore compared the receptor distributions between turkey and chicken tissues. Using lectins which bind preferentially to 2-3Sia or 2-6Sia, we found that turkeys have a greater abundance of 2-3Sia in the nasal turbinates and trachea, comparatively higher than those found in equivalent chicken tissues. The nasal turbinates and trachea are the major sites of H7N9 replication, and therefore might explain the rapid emergence of the L217Q polymorphism reported previously [24]. However, analysis of the turkey visceral organs revealed comparatively lower 2-3Sia levels than 2-6Sia, as observed by others [47], suggesting that differences in receptor distribution alone are not likely responsible for the increased tropism or observed pathogenicity. Receptor distribution in chicken and turkey tissues performed by lectin binding served to assess the overall distribution of the 2-3Sia and 2-6Sia, and did not identify other receptor derivatives, such as those possessing specific modifications, e.g. sulphated 2-3Sia which appear to be important as receptors for other AIVs [48,49]. It has also been recently reported that the abundance of N-linked glycans on the cell surface may also influence the receptor-binding preference of AIVs [50]. Therefore, a more detailed investigation into the glycome of different avian species is required to fully explore this mechanism, and its possible role in effecting H7N9 LPAIV binding to different turkey tissues, and how this may differ for the H7N9 wt and ty-ad LPAIV variants.

We also compared the replication kinetics of both the *wt* and ty-ad viruses in a range of avian and mammalian cells. In turkey, chicken and duck primary cells, the ty-ad virus showed a statistically significant increase in the level of replication compared to the *wt*. However, no statistically significant differences in replication were observed in either human, swine, or the highly sensitive and permissive MDCK (canine) cell line. Together, these observations indicated that the ty-ad virus confers a generic replicative advantage in avian but not mammalian cells. This similar level of replication in mammalian cells with Q217 has also been previously observed [14]. Clearly, multiple factors determine the replication kinetics of AIVs. Considering that the polymorphisms in the ty-ad virus are outside the polymerase, the dynamism of vRNA replication is unlikely to be a contributory factor. This observation is more likely due to different receptor utilization, or possibly related to a different pH of HA activation. The optimal pH of HA activation, or pH of fusion, is species dependent, and pH has been demonstrated to alter replication kinetics in ex vivo organ cultures [51] and for an H5N1 HPAIV isolate *in vivo* [52]. Q217 slightly decreases the pH of fusion when assessed by syncytia formation assays [14]. Interestingly, it has been suggested that AIVs tend to favour a lower pH of fusion upon infection and circulation in turkeys in comparison to those isolated from other avian hosts [53].

Another consideration, not explored in this study, is the differential host response induced by the *wt* and ty-ad viruses. Indeed, turkeys appear to mount a different host response than chickens in response to H7 infection, particularly from genes involved in RNA metabolism and the innate immune response [38]. However, it is uncertain whether single amino acid changes in the HA and NA proteins would contribute directly to different immune responses or enhance immune evasion of these viruses. The immune response to IAV infection in different avian species, including different galliforme species, is understudied and requires further investigation.

Alongside the Q217 polymorphism identified in the ty-ad virus, a threonine-to-isoleucine polymorphism was also detected in the NA protein at residue position 10 (T10I). The function of this mutation has not been documented in the literature. However, this polymorphism is located in the transmembrane domain of NA, away from the functional site and stalk, hence this polymorphism is unlikely to contribute to the different pathogenicity observed across the two species.

Antigenic comparisons between viruses possessing either Q217 or L217 induced 23- and 8-fold reductions in HAI titre with a ferret and chicken antisera, respectively [54]. In this study, we saw similar antigenic differences between the ty-ad (Q217) and *wt* (L217) H7N9 viruses, reinforcing this finding. However, the differential receptor binding of the L217Q mutation has been postulated to reduce the avidity of binding to chicken red blood cells, thereby overexaggerating antigenic changes in HAI assays [12]. Despite the possible effect of different avidities, the L217Q polymorphism has previously been shown to emerge as an antigenic ‘escape’ mutant upon incubation with H7N9 A/Anhui/1/13 antisera, indicating a genuine role of Q217 in the HA antigenicity of H7N9 [54]. In addition, since 2017, China has implemented a mass vaccination programme for poultry against H7N9. This campaign has successfully reduced zoonotic cases yet has not eradicated H7N9 from poultry in China. Genetic analysis of H7N9 viruses detected in poultry after the introduction of the vaccine has highlighted an increased detection of Q217 in H7N9 HA gene sequences [10,11]. The vaccine consists of the HA antigen derived from an A/Anhui/1/13-like candidate vaccine virus [54]. As such, this work suggests that Q217 emergence through antigenic evolution may afford greater pathogenic outcomes in some susceptible avian species, such as turkeys. Through *in vitro* characterization, Q217 appears to have reduced zoonotic ability [14]. However, in this study, we observed that the ty-ad virus was still capable of infecting ferrets, producing similar levels of vRNA in nasal wash sample when compared to H7N9 *wt* virus ferret infections conducted in the same animal facilities [22,34]. This observation suggested that while the ty-ad virus possesses no greater zoonotic risk to *wt*, it still likely retains its ability to infect humans. We also explored the potential for this virus to spread from infected ferrets to chickens or turkeys, in an attempt to mimic reverse zoonotic transmission. In this study, infected ferrets were physically separated from chickens and turkeys but shared the same airspace for two 6 h periods. No evidence of airborne transmission to naïve birds could be detected. While longer or more intermate contact may have facilitated successful transmission, these results suggest that the ty-ad H7N9 does not exhibit a strong ability for reverse zoonotic transmission. However, infection spread among avians included the extension of the successful chicken transmission chain to a second contact group (R2) of chickens. Daily refreshing of the drinking water and changing of the bedding at 4 dpi suggested that spread from R1 to R2 chickens did occur, with the detected viral environmental contamination by both wt and ty-ad viruses potentially contributing to the acquisition of infection, as described elsewhere [56].

In conclusion, this study has demonstrated that the ty-ad virus emerged rapidly following infection of turkeys, possibly due to high levels of 2-3Sia in the major sites of virus replication in turkeys. When this adaptation occurred, it enabled the virus to replicate systemically in a wider range of organs, and at implied higher titres. This enhanced tropism conferred increased pathogenesis and mortality on infected turkeys. The enhanced tissue tropism was not due to any change in the ability of the virus to replicate without trypsin or to any obviously different receptor distributions in the turkey organs. However, the adaptation did confer a statistically significant increase in replication in avian cells, including turkey cells, but not in mammalian cells. The exact mechanism for the systemic tropism of ty-ad virus and its associated pathogenesis in turkeys remains elusive. However, this work highlighted the requirement for continued surveillance of low-pathogenicity H7N9, particularly as vaccination programmes continue.

## supplementary material

10.1099/jgv.0.002008 Uncited Supplementary Material 1.

## Abbreviations

ANOVA: analysis of variance
AUC: area under the curve
BHIB: brain heart infusion broth
cDNA: complementary DNA
CEF: chicken embryo fibroblast
CS: cleavage site
DEF: duck embryo fibroblast
DMEM: Dulbecco’s Modified Eagle Medium
dpi: day-post-infection
EFE: embryonated fowls’ egg
EID_50_: 50% egg infectious dose
EP: egg passage
ETE: embryonated turkeys’ egg
HA: haemagglutinin
HAI: haemagglutination inhibition
IAV: influenza A virus
IHC: immunohistochemistry
IV: intravenous
IVPI: IV pathogenicity index
LPAIV: low-pathogenicity avian influenza virus
MALII: *Maackia amurensis lectin* II
MBCS: multi-basic cleavage site
MDCK: Madin-Darby canine kidney
NA: neuraminidase
PB: basic polymerase
r.e.u.: relative equivalent unit
rRT-PCR: reverse transcription real-time PCR
SA: sialic acid
2-3Sia: α2-3-linked sialic acid
2-6Sia: α2-3-linked sialic acid
SNA: *Sambucus nigra*
TEF: turkey embryo fibroblast
TPCK: L-1-tosylamido-2-phenylethyl chloromethyl ketone
ty-ad: turkey-adapted
vRNA: viral RNA
WGS: whole-genome sequencing
WOAH: World Organisation for Animal Health
wt: wild-type
