# Supplementary material for: Evaluating the epizootic and zoonotic threat of an H7N9 low-pathogenicity avian influenza virus (LPAIV) variant associated with enhanced pathogenicity in turkeys
Source: J Gen Virol. 2024 Jul 9;105(7):002008. doi: 10.1099/jgv.0.002008 (PMC11316556; doi:10.1099/jgv.0.002008)
Supplement: Uncited Supplementary Material 1. [file jgv-105-02008-s001.pdf]

Fig. S1

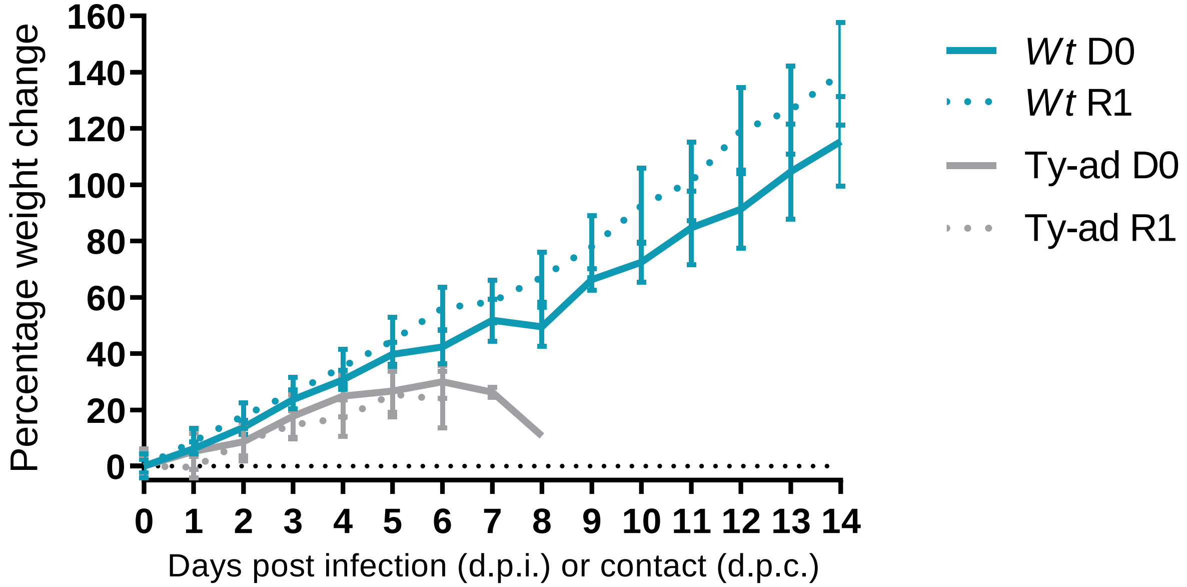

Fig. S1. Percent weight change of turkeys infected (D0) with *wt* or ty-ad virus variants, or of turkeys housed in contact (R1) with either group.

Six turkeys (D0) were infected via the oronasal route with $1x10^6$ EID_{50} or either *wt* or ty-ad virus variants. Six further turkeys (R1) were co-housed with either group at 1 day post infection. All turkeys were weighted daily, and percentage weight change was calculated from their original weight prior to infection. Dotted horizontal line indicates zero percent weight change.

Fig. S2

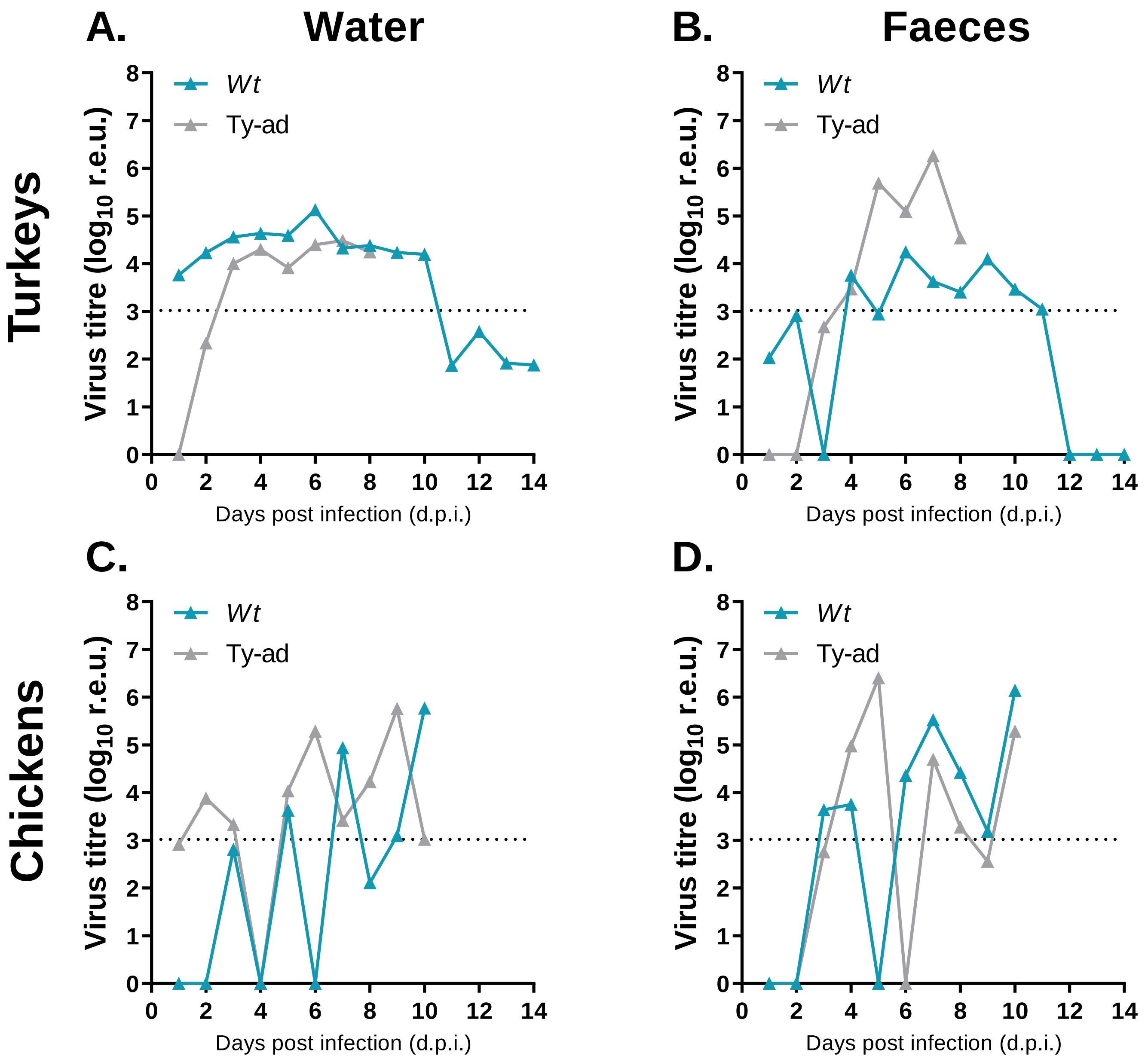

Fig. S2. Detection of vRNA in environmental drinking water and faecal samples following infection and transmission of *wt* or ty-ad virus variants in turkeys and chickens.

vRNA detection in drinking water (**A and C**) or faecal samples (**B and D**) collected from the environment of infected or contact turkeys (**A and B**) or chickens (**C and D**), from the experiments shown in **Fig 2** or **Fig 3** respectively.

Fig. S3

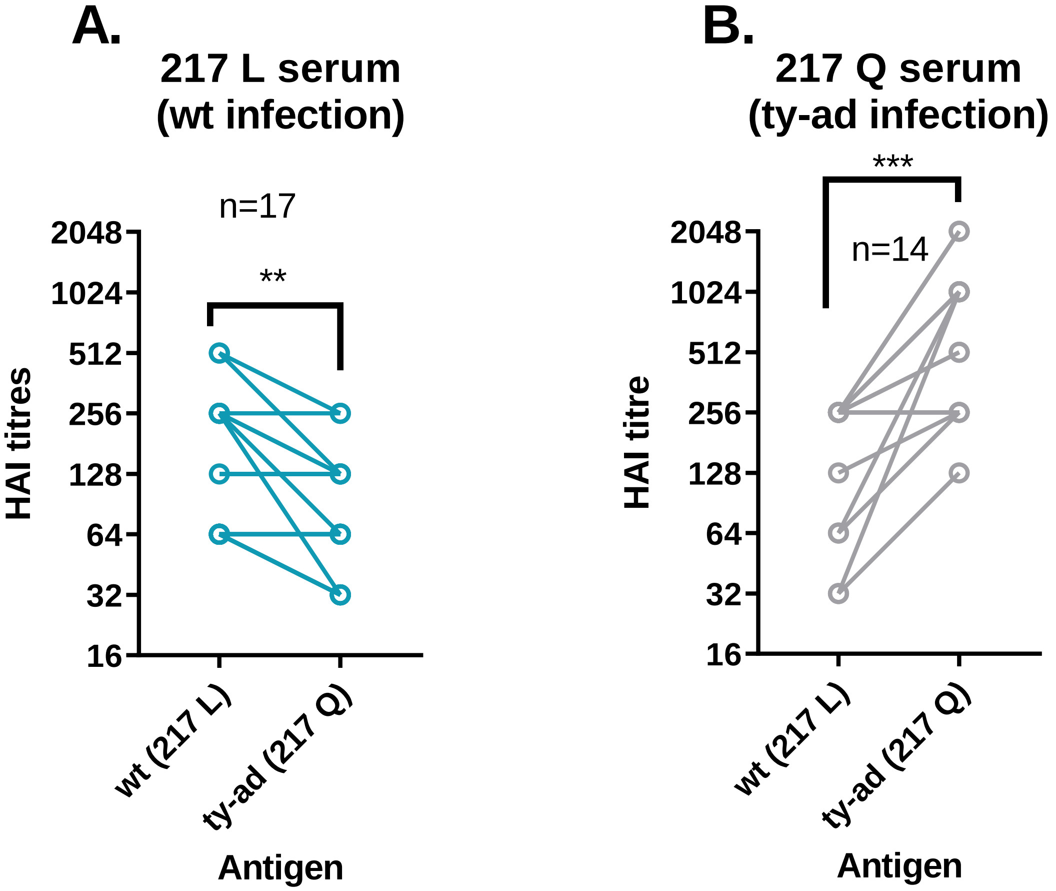

Fig. S3. Homologous and heterologous H7 HAI titres from infected turkeys.

HAI assays were performed on paired sera taken from chickens and turkeys, following initial infection via the ocular-nasal route with (**A**) *wt* (217L) (total n=17; n=8 chickens; n=9 turkeys) or (**B**) ty-ad (217Q) (total n=14; n=6 chickens; n=8 turkeys) viruses. Sera were collected between 8- and 19-days post infection. The HAI assays were performed using homologous (e.g. *wt* sera and *wt* antigen) or heterologous (e.g. *wt* sera and ty-ad antigen) antigens. Individual points are shown with lines connecting paired sera. Paired t-tests were performed comparing the HAI titres of individual sera tested using the wt and ty-ad antigens. *** indicate p-value <0.001. ** indicate p-value <0.01.

Fig. S4

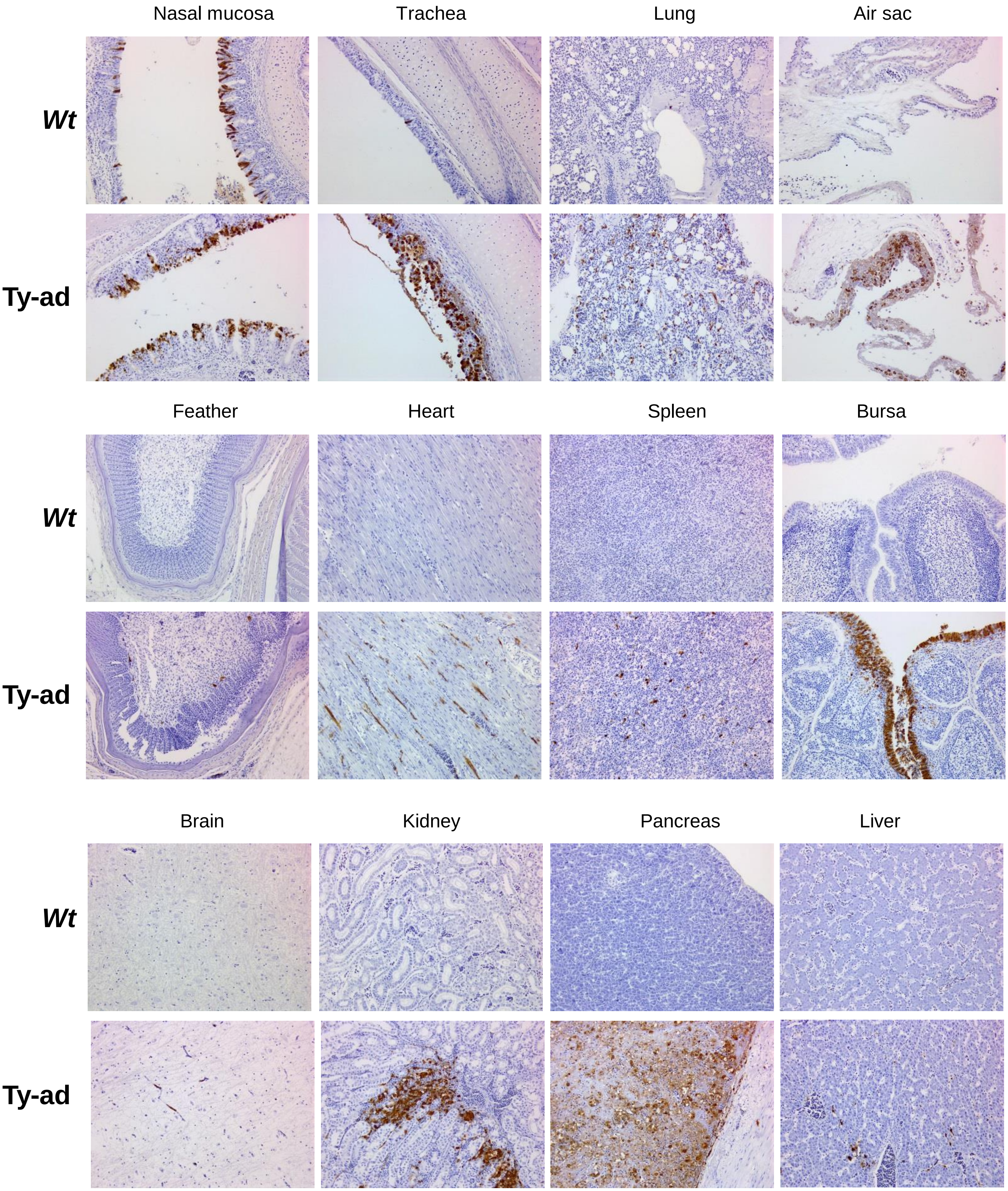

Fig. S4. Representative IHC images from two turkeys infected with *wt* or ty-ad H7N9 viruses.

Representative IHC images showing the presence of influenza A virus Nucleoprotein (NP) antigen on the indicated tissues collected from turkeys infected with *wt* or ty-ad H7N9 viruses. Tissues were collected from birds (*wt*, bird E or ty-ad, bird G) which were culled at 6 dpi. NP labelling is shown in brown.

Fig. S5

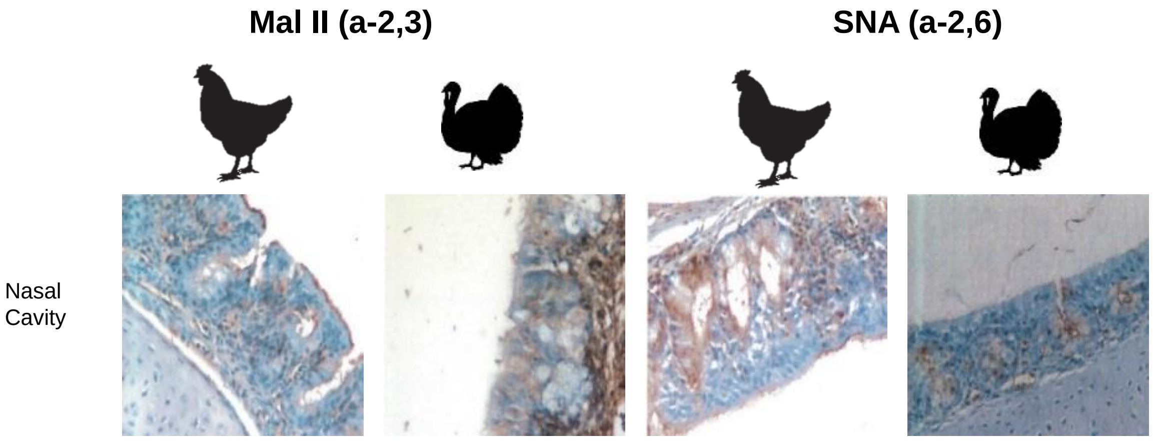

Fig. S5. Representative images showing lectin staining for sialic acid receptor distribution in chicken and turkey tissues.

Representative images showing the staining of α2,3 (Mal II) and α2,6 (SNA) sialic acid using lectins. Respective lectin staining of tissues collected from the nasal cavity of uninfected chickens and turkeys shown in brown.

Fig. S6

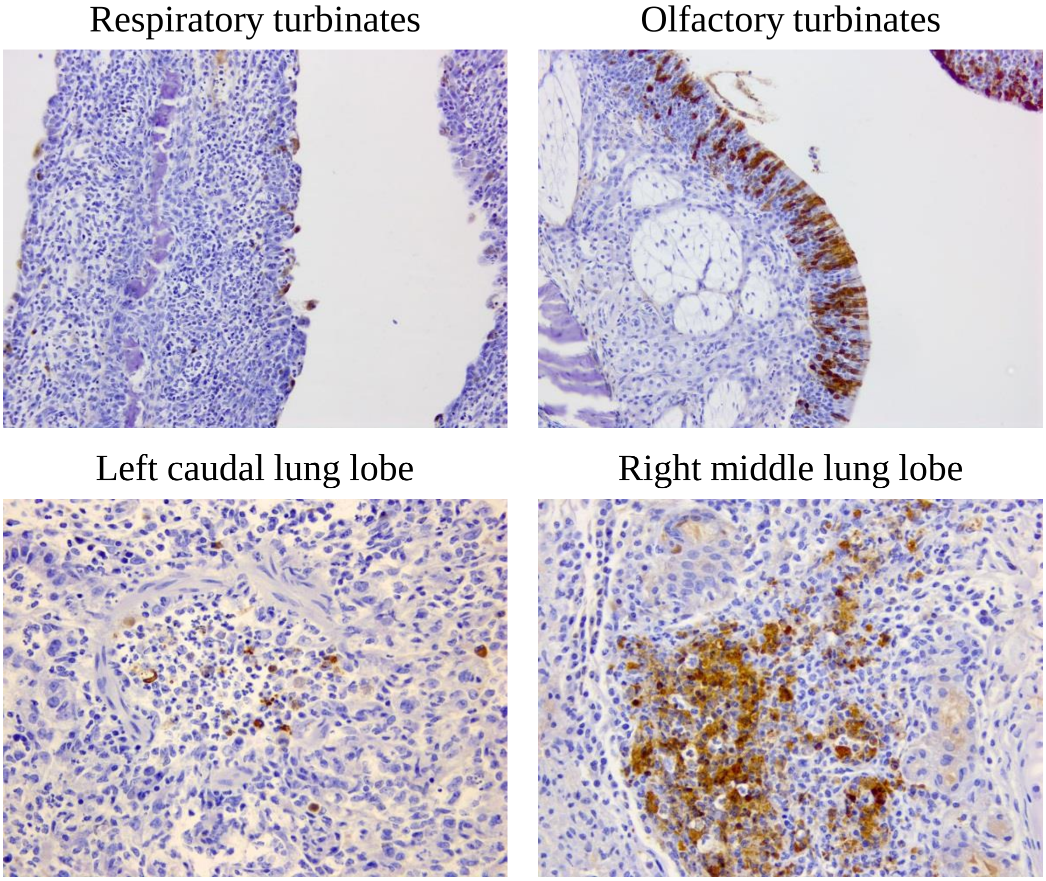

Fig. S6. Representative IHC images from two ferrets infected with *wt* H7N9 viruses.

Representative IHC images showing the presence of influenza A virus Nucleoprotein antigen in the indicated tissues collected from ferrets infected with the ty-ad H7N9 virus. Tissues were collected from ferrets which were culled at 6 dpi. NP labelling is shown in brown.

Table S1

Table S1. Semi-quantitative evaluation of the influenza A type-specific NP antigen labelling by IHC. H7N9 LPAIV (wt and ty-ad) tropism was investigated in turkey tissues, following culls at 4 and 6 dpi when apparently healthy.

	4 DPI				6 DPI			
	Wt		Ty-ad		Wt		Ty-ad	
**Bird ID**	**A**	**B**	**C**	**D**	**E**	**F**	**G**	**H**
**Heart**	-	-	++	+/-	-	-	+	++
**Skin**	-	-	+	-	-	-	+/-	+
**Feather follicles**	-	-	+	-	-	-	+/-	+
**Skeletal muscle**	-	-	+	-	-	-	+/-	+
**Spleen**	-	-	++	+/-	-	N/a	+	++
**Brain**	-	-	+	-	-	-	+	++
**Kidney**	-	-	+	-	-	-	+	++
**Ovary / Testes**	T -	T -	N/a	O -	O -	N/a	O +	O ++
**Cecal Tonsil**	-	-	+	-	-	-	+	+
**Thymus**	-	-	+	-	-	-	+/-	+/-
**Bursa**	-	-	+	-	-	-	+++	+++
**Lung**	-	-	+++	+	-	+/-	+	++
**Trachea**	-	-	+++	+	-	+	++	+++
**Air Sacs**	-	-	+++	-	-	-	+++	+++
**Nasal cavity**	-	+++	+++	+	++	++	+++	+++
**Pancreas**	-	-	+++	-	-	-	+++	+++
**Duodenum**	-	-	+	-	-	-	+/-	+
**Liver**	-	-	++	+/-	-	-	+	++
**Proventriculus**	-	-	+	-	-	-	+/-	+
**Jejunum**	-	-	+	-	-	-	+/-	+
**Colon**	-	-	+	-	-	-	+/-	+
**Caecum**	-	-	+	-	-	-	+/-	+

O, ovary; T, testes; N/a, not available; -, absent; +/-, minimal; +, mild; ++, moderate; +++, diffuse

Table S2

Table S2. Semi-quantitative evaluation of the influenza A NP antigen labelling by IHC in tissues collected from ferrets at 6 dpi following infection with the ty-ad H7N9 variant.

**Tissue**	**Detailed anatomical location**	**Ferret ID**	
		**1**	**2**
Turbinates	Nasal turbinate	+	+
	Olfactory turbinates	++	+/-
Glands	Salivary gland	-	-
Trachea	Cervical trachea	+/-	-
	Thoracic trachea	+/-	+/-
Lung lobe	Right cranial	-	-
	Right middle	+/-	++
	Right caudal	-	-
	Accessory	-	+
	Left cranial	-	-
	Left caudal	-	+/-
Lymph Nodes	Mandibular	-	-
	Retropharyngeal	-	-

-, absent; +/-, minimal; + mild; ++, moderate; +++, diffuse.
